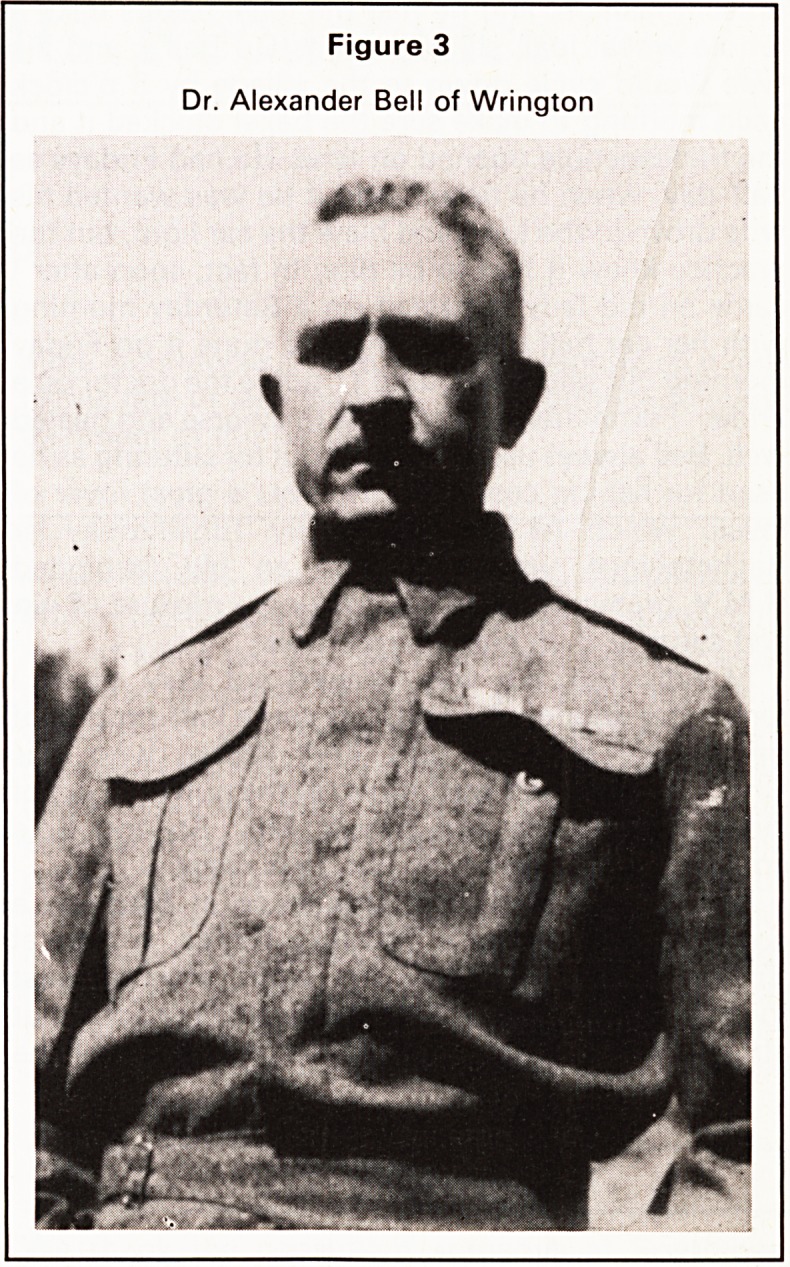# Man and Medicine on Mendip

**Published:** 1984-04

**Authors:** Norman Tricks

**Affiliations:** General Practitioner, Wrington


### Man and Medicine on Mendip

### Presidential Address delivered to the Society on 12th October 1983

**Norman Tricks**
General Practitioner, Wrington

I would first like to thank you for installing—I can hardly say electing—me into this honourable office. In selecting a rural G.P. I wondered if you hoped that the dung on his boots would help fertilise this old society. When Dr. Joe Cates proposed me last year for this office, he referred to that illuminating presidential address of my erstwhile senior partner, Dr. Gordon Heron, entitled 'That Backbone' (i.e. general practice).

The backbone I am going to talk to you about is the great backbone of North Somerset—Mendip. It starts near Frome and extends north-westwards with many caudal vertebrae ending at Brean Down and Steep Holm in the Bristol Channel.

I was fortunate enough to grow up as a boy at Blagdon, to live at Rowberrow as a medical student, and then to come back to practise at Wrington and Blagdon. Mendip, is a land of contrasts, part of our practice in Churchill is below high tide level and part at Charterhouse over 1000 feet. There are the great gorges of Cheddar and Ebor, the gentler lovely coombes such as Goblin coombe, Chelmscoombe, Rickford and Burrington, with the famous Rock of Ages, where Toplady, the curate of Blagdon, wrote his well known hymn 'Rock of Ages', and the shallower bottoms of Velvet Bottom, Long Bottom and Lambsbottom. There are the windswept uplands with lovely straight roads and the dry stone walls and the barrows on the hilltops as at Priddy.

The southern slopes have their horticulture and their caves, while from the northern slopes issue a succession of springs giving rise to the beautiful man made lakes of Chew, only created 30 years ago, and Blagdon, now over 80 years old.

Now for a brief resumé of Mendip history, beginning with the Stone Age. The first known Man on Mendip survives as the skeleton of a young man of 23 found in the silt of Goughs cave where he probably drowned in a flood. He was 5′4″ tall, slender, long headed with flattened leg bones, possibly due to squatting—although this is not noted in the women of Mendip. However, skeletons in East Anglia of the same era show the women with flat leg bones and not the men! Over 50 skulls and implements of this Stone Age were found in Avelines hole in Burrington, they were coated with stalagmite forming a perfect cast of the veins, but unfortunately they were destroyed in the air raid on the University.

About 1800 B.C. the Beaker folk came to Mendip, they were round headed and were buried in round barrows and got their name from the beaker-shaped cups they used. They built the henges—like Stonehenge, Avebury, Stanton Drew and Charterhouse. The Beaker folk were superseded about 1700 B.C. by the Bronze Age race who cremated their dead and buried the bones in bowl-shaped barrows—good examples are those of Priddy and Blackdown.

The Iron Age people came next and they left us with that great impressive camp at Dolebury, near Churchill. Its ramparts are $\frac{7}{8}$ of a mile round and enclose 20 acres with a double line of fortifications and when you see the size of the walls now and consider how they must have been eroded by weather over the 2000 years, you realise what a task they were to build. There is an old proverb 'If Dolebury digged were, Gold would be in share'. It has just been acquired by the National Trust.

With the Romans came Mendip's great prosperity as it was the centre of lead mining. This lead was sent all over the Roman world along the Roman road which crossed Mendip from Uphill to Salisbury. The earliest known pig of lead in Britain, dated A.D. 49, was found at Blagdon and the heaviest, 223 lbs., at Charterhouse where the Romans had a settlement (Figure 1).

After the Romans there was a gap in the mining records until the reign of Edward IV when the

**Figure 1**

Lead works at Charterhouse

Mendip Mining Laws were framed, some of which are aimed at limiting pollution and its dangers.

We are reminded of the old mine workings at Charterhouse in Macaulay's poem 'The Spanish Armada'—'The rugged miners poured to war from Mendip's sunless caves'.

The coming of the enclosure act about 1770 removed the need for these mining laws, but led to the establishment of farms, as at Nordrake, near Charterhouse. Here lived a lovely old man, called 'Farmer', who was a great authority on Mendip. He came to see me once because he was giddy and asked if he could drive. After much discussion we agreed that he could only drive on Mendip top, but as he went out of the door he said 'if I were 10 years younger, Zer, I would jump up on the old horse instead of driving him'. When he died a few weeks ago they were worried where he had hidden the money as they only found £1000 in one boot and a bottle of whiskey in the other, and a sack of half-crowns in the cowshed.

When the lead mines were closed because of the expense of deeper mining then calamine mining started, especially in Shipham and Rowberrow. This was used for the production of brass and zinc, for which there was a ready market in the brass works in Bristol. These calamine mines left large areas called 'gruffy ground'.

The locals knew of this contamination of the soil and it was described by Frances Knight in his book 'A corner of Arcady'—when he built himself a house at Shipham. They said 'us told ee zo, tis that there minedry, you won't never be able to grow nothing there'. In January 1979, when Shipham suddenly hit the headlines with titles like 'Doomwatch village' and 'Poison Village' many of the older inhabitants were very sceptical and there was quite a lot of resentment. It was all due to a report of a survey by Imperial College, London, on the cadmium content of the soil and was published without consulting local doctors or the parish council. We all thought that Shipham residents were very healthy and probably lived longer than average, and a few sceptics even suggested the sensation was released that day to mask some unpalatable financial news by the government—and this it certainly succeeded in doing. Some newer inhabitants worried, probably more from the value of their properties than their health, and the papers carried headlines for the next few days like 'Don't panic Shipham'. *The Observer* had a long article comparing it to Itai-Itai disease, of which there was an outbreak in Japan due to contamination of rice by cadmium.

With a great flurry of activity, a survey committee was set up and the residents subjected to various tests. The outcome, after an expenditure of £200,000 and two years work, was that there was no apparent adverse effect on health but local grown leaf crops such as cabbage, spinach and lettuce should be avoided. The village flower show even produced a cup for the best vegetables called the 'Cadmium Cup' and the pub produced a cocktail the colour of cadmium.

Mendippers are reluctant to deal with officialdom but sometimes they can turn it to their advantage: I remember I had a case of brucellosis, common with us then, and we traced the source to a farmer who was told by the Health Inspector to stop selling untreated milk—whereupon he asked him to 'Give I a sustificit to the effect that I's milk do cause abortions, because then it be worth £1 a pint in Bedminster'.

Rowberrow has a couple of medical slants. First Rowberrow was the home of the Brett Young family and if you have read Frances Brett Young's book 'The Young Physician' you will recall how as a medical student he came back to visit his grandmother, who lived there. Rowberrow also has a Saxon Cross of about 709 A.D. now in the church, which depicts a long serpent (about 18 ft. long) entwined on a rod, perhaps an example of the Staff of Aesculapius. The Registers here contain some curious names given to children: one boy is called 'Anon' and three girls are called 'Talitha Cumi' which is the expression used by Christ at the raising up of Jairus' daughter and means 'Maiden, I say unto you arise'.

We are very fortunate that the diaries of two Mendip doctors of the late 1600's have been preserved, Dr. Claver Morris and Dr. John Westover.

Dr. Claver Morris of Wells, lived from 1659 to 1726. He graduated from Oxford and came to Wells in 1686. His diaries make fascinating reading about the life of those times, but he was very discreet and gives little clinical detail about his patients or his prescriptions. He had a laboratory and records many hours working in it compounding new medicines. He seems to have been more like a consultant physician and was often called in by other doctors or apothecaries, although he was often critical of their dosage. He deplored 'languid medicines' and advocated large doses. His diaries contain many hieroglyphics.

Dr. Morris travelled vast distances for consultations, to Exeter, Bath and into Gloucestershire, and often he describes difficult travelling conditions—once lost in fog on Mendip top, once caught in a bog at Charterhouse, once with the snow up to the belly of his horse and once his horse being frightened by a bear. He was a great socialite as well as a Commissioner for Sewers and Taxes, and describes his entertaining and public duties. He was a great lover of music and although he usually started the day early and went to service in the Cathedral at 6 he often ended up with a Consort of Music and did not get home until the small hours. These consorts were at first held with the full moon, presumably so that people could see their way home, but later became more frequent and he often describes what and how they played. He also describes how the Bishop of Wells, Bishop Kidder, and his wife were killed in a hurricane when the palace chimney blew down on them while in bed.

Dr. John Westover of Wedmore lived from 1643 to 1706. The doctors 'plate' is still attached to his house in Wedmore. His practice extended to Langford, Wrington and even Bristol. He came in a long line of John Westovers, seven in all, and he took over his father's practice just after Judge Jeffrey's bloody assize. His daybook from 1686 has fortunately been deciphered by the Rev. Sydenham Harvey. It is divided into three parts: the first is an account of his every day dealings with his patients. In this book are set down all the ailments, agues, distractions, dislocations, fractures, fevers, jaundices, melancholies, pains, swellings, stitches, itches; all the cordials, carminatives, decoctions, electuaries, dyet drinks, juleps, marmalades, opiates, pills, potions, sudorifics, cephallicals, pectorals and stomachicals which they swallowed, all blisters, plasters, poultices and cataplasms applied and all the teeth and all the fees which were extracted from them. The second deals with inpatients (of which more anon) and the third with his farming interests. Like all country G.P.s, even now, a little farming on the side was common—it keeps your hand in with midwifery. Looking at extracts from the day-book, if two figures are given for the year it is because the year then did not start until 25th March, in fact Rowberrow Church has a tombstone of a child born in April and dying in February of the same year—1751.

One entry states 'Cousin George Counsell had a girdle for the itch'. Westover very commonly used a girdle, in fact a girdle had been in use for various complaints since mediaeval days. I found in a book on Mediaeval medicine the story of a knight called Humphrey who, in 1095, suffered from severe dropsy. He was given a girdle which belonged to St Anselm, Archbishop of Canterbury, and this was wrapped tightly round his limbs until they were relieved—perhaps an example of compression technique. A miraculous cure was attributed to the girdle and perhaps this is how the superstition was carried down.

The second part of his daybook deals with the inpatients, those who came to 'cure' and those who came to 'Table', that is those he thought he could cure and those who were deemed incurable, usually because of madness. He housed these patients in a 'hospital' next door to his house (Figure 2) and this was probably the first private psychiatric hospital in the country.

This hospital still stands today and has recently been beautifully restored and converted to a lovely house by Norman Frost, whom I expect many of you will remember as the Chief Constable of Bristol. When he bought it, it was in a derelict state and the lockers for the patients were still in existence in the dormitory on the third floor, the middle floor being used as a day room and the ground floor as a stable.

**Figure 2**

Dr. Westover's Hospital at Wedmore

The third part of the daybook deals with his farming affairs and is even more intriguing. Here is one entry referring to medicinal flowers: 'Richard Adams and I came to account, he being paid for his Hisope and Clove Gilley flowers, just 3s 3d'. These were used for fevers and strengthening the heart. We will finish John Westover appropriately with the epitaph on his father's grave which reads:

Here resteth the body of
JOHN WESTOVER,
Chyrugion

Who departed This Life
January 30 1678

"Of Death Let This A Warning Be
Unto Such As Pass By
Expect A Sudden Change To See,
Repent, For Doctors Dye".

It may surprise you, as it did me, to discover that Wrington had its own hospital, established in 1864 with Dr. Horace Sweet as the local medical officer and an honorary staff of doctors from as far away as Weston, Clevedon, Axbridge and Chew Magna. The preamble to the rules says 'the patients will have the advantage of the combined skills of several practitioners in severe cases or accident'. It was pointed out that the patients were loth to travel to town hospitals and that the journey, even if fit, was an ordeal of jolting and that 'there was a danger of erysipelas ensuing from the atmospheric influences of large towns'.

The cost of establishing the hospital, which had 5 beds, was £92 9s. 5d. including the beds at £14, ironmongery £6 and medical stores £4. The first year 24 patients were admitted, 18 cured, 3 relieved, one incurable and two died. The average stay was 35 days. The running costs for the first year were £102 19s. 4d., of which nursing was £28, wine and beer £5 and medicine and surgical appliances £2 10s. 0d. This left a balance in hand of £11.

The next report still in existence is of the third year, 1866. The main change was the resignation of Dr. Sweet and the appointment of Dr. George Barnes after references from various Harley Street and Charing Cross consultants. This was another favourable year with a balance of nine shillings and tenpence. Although beer and wine had gone up to £6, medical supplies had dropped to £2 0s. 9d. For some reason the hospital closed about 1870 and when, a few years later, it was proposed to open another, it was stated that 'the first one closed neither because of shortage of money, or lack of patients'—in fact there was a balance which is still invested for our district nurses to this day.

**Figure 3**

Dr. Alexander Bell of Wrington

I would like to tell you for a moment about my predecessor in my practice who to one or two of you will be almost a hero, but the majority of you will have never heard of him and only a few will have met him because he was a very shy retiring man. However, to a great band of men throughout the country he will be more famous than Pasteur, Curie or Fleming—I refer to Dr. Howard Alexander Bell (Figure 3). And why, you will say, was he so famous? I quote from his obituary in the *Journal*—which *Journal* you say? 'It is sad to record the death at 86 of Dr. Bell of Blagdon and Wrington who conducted over 3,000 autopsies'. Now you may think this is an awful lot for a small practice and he must have been a terrible doctor, but the bodies he was examining so carefully were fish, for it was Dr. Bell who revolutionised the method of fly fishing for trout. By careful observation of their habits and by all these autopsies and by great skill in trying new types of fly he revolutionised the sport. I won't go into details, as those of you who fish will know about him and those who don't won't be interested. His goldfish pond was a replica of Blagdon Lake and his weather vane was a salmon. He was a great countryman and a great doctor with great powers of observation. He did not agree with chairs in his consulting room because he said if patients sat down they stayed and he always stood himself, writing on a cupboard top, but he always listened very carefully to the history, for he said if you can't tell what is wrong after five minutes history you are no damn good. When he retired at 75 I, as a youngster, thought I would find many mistakes, but I never found any and yet he never went to any meetings. In fact he never left the practice area, but always noted very carefully anything in a consultant's letter.

He was very autocratic, although shy, and had very few friends. If he thought there were too many in my surgeries he would send them away and they went meekly. He disapproved of street names, saying they weren't necessary in a village and gave the lads 2/6d. if they pulled down a street name. He was also long before his time in the advocacy of a high-fibre diet. All patients were instructed in this long before the present fashion, and he had the local bakers bake a whole wheat loaf, still known as Dr. Bell's, and his wife would cycle down to the village by 8 o'clock each morning to make sure the baker stocked it and the tradespeople opened on time. He had Fridays as half-day, when he fished, and if he was wanted his wife drove to the Lake and blew the car horn, but the practice knew it had to be dire. In fact, soon after I came an old lady appeared on a Saturday morning with her ear half cut off. She had done it on Friday morning but said she couldn't trouble the doctor on a Friday. Fortunately it was none the worse and healed well. Bell always used the same gut for suturing as he used for fishing casts. He also was a great lover of music which he played, usually Tchaikovsky or Rachmaninof, very loudly on an old fashioned H.M.V. gramophone while drinking a mixture of gin and syrup B.P. in equal proportions.

Mendip folk are very shrewd yet very open. I remember asking why one man was a half-brother of one farmer and was told 'Well Father, who was Churchwarden, did walk down to the pub for a jug of cider most nights and passes Mrs. So and So's cottage, so that was how our Jack came'.

Recently I found in a farmhouse at Blagdon a copy of John Wesley's 'Primitive Physic' written at Bristol in 1755. He uses the word 'tried' against those remedies which he thinks most efficacious, but above all extols the virtue of 'electricity' which he says 'comest nearest to a universal medicine as any in the world.'

This old farmer where I found the book still believed he could cure his rheumatism by changing his cider from Draycott to Blagdon and his wife asked 'how it was down to Wrington where all the plagues came from?'

Mendippers are also resourceful. Only last week a farmer badly cut his hand with a billhook, so nipped round to the farm next door and took the skin closure strips off the forehead of his nephew, who had recently had a road accident, and stuck them on his own hand.

But above all I think Professor Richard Doll would be pleased with the story of the Butcombe farmer who said to me 'You know doctor, our brother Lewis, who be half a prophet, do reckon it bain't the cigarettes which do 'ee 'arm—no, he do reckon it be the baccy they puts in 'em'.

So I must finish, it has been rather a ramble, but that is what Mendip is for, rambling.

I hope those of you who don't already know it will be encouraged to ramble over it and many of you will call in on us at Wrington.

As the story goes on Mendip of two men meeting: 'where be 'ee going George?' says one, and gets the reply 'Bain't be going nowhere, be coming back'—that's really what I feel like.

Thank you for coming. Thank you for listening.

## Figures and Tables

**Figure 1 f1:**
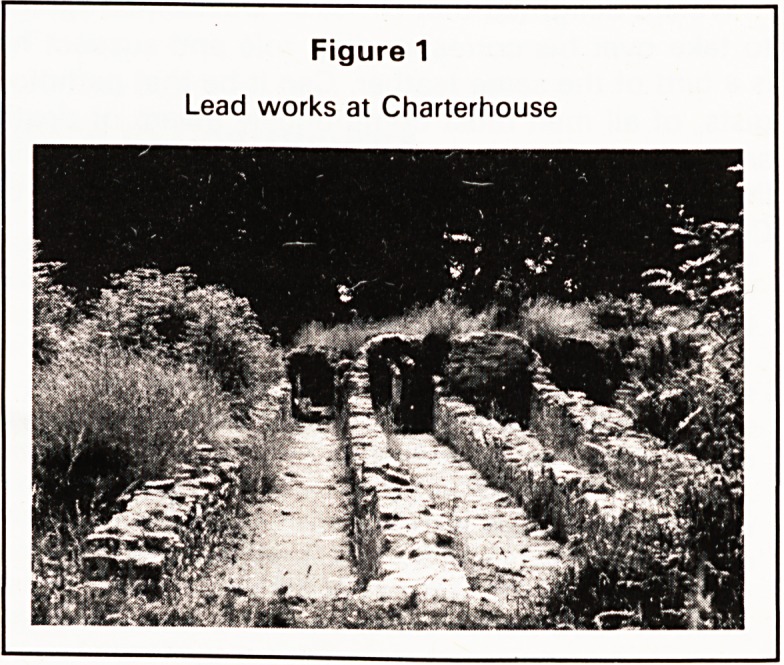


**Figure 2 f2:**
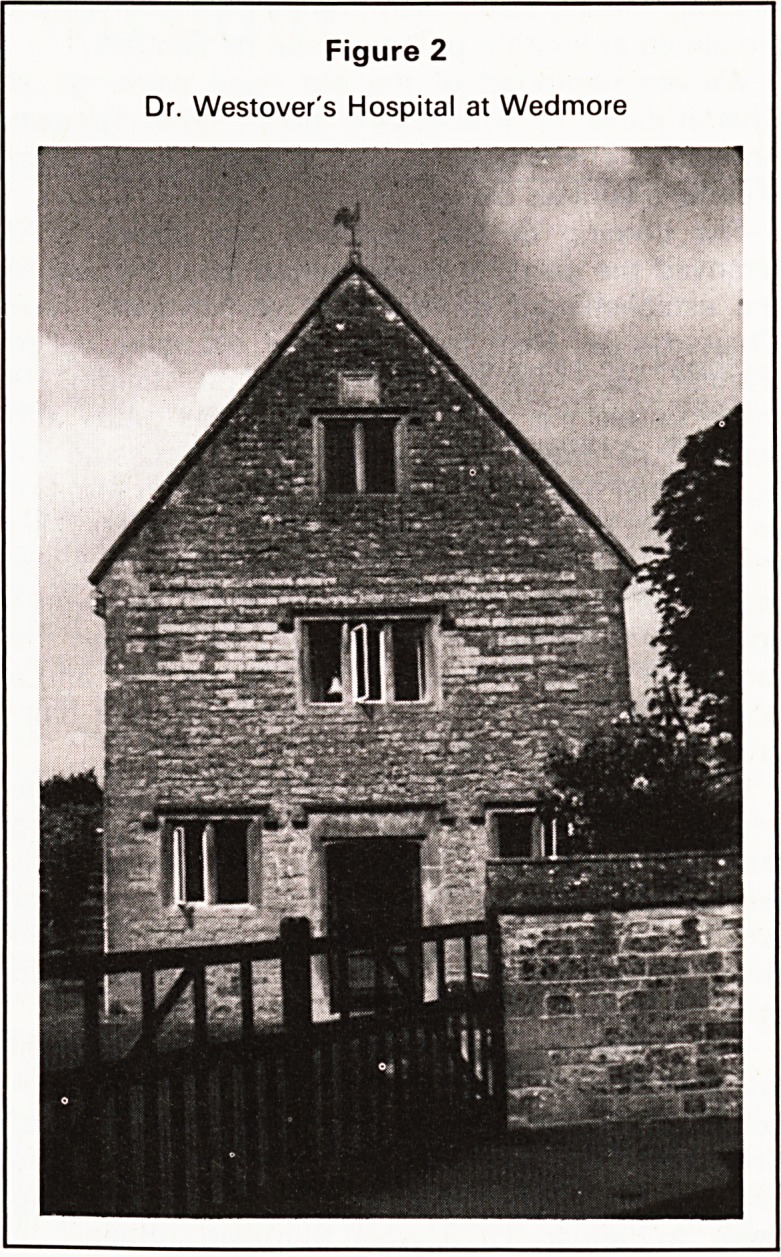


**Figure 3 f3:**